# Supplementary material for: m6A Demethylase ALKBH5 Restrains PEDV Infection by Regulating GAS6 Expression in Porcine Alveolar Macrophages
Source: Int J Mol Sci. 2022 May 31;23(11):6191. doi: 10.3390/ijms23116191 (PMC9181496; doi:10.3390/ijms23116191)
Supplement: Supplementary file 1 [file ijms-23-06191-s001.zip › ijms-1702786-supplementary.pdf]

Table S1 Primer information for RT-PCR identification

Primer name	Sequence of the primer	Length of products (bp)
PEDV-M	F: 5'- TCCCGTTGATGAGGTGAT- 3'<br>R: 5'- AAGCATTGACTGAACGACC- 3'	551
PDCoV-N	F: 5'- CCAAACGCAACCCCAACAATCC- 3'<br>R: 5'- CTTCTCAGTGTCTGCAGAGCCG- 3'	329
PoRV -VP6	F: 5'- CAAACGGGAGGAATAGGAA- 3'<br>R: 5'- CACTCTTGGGAAACTGAACC- 3'	572
TGEV-S1	F: 5'- CCAAACAGCCGTTATTAGTTA- 3'<br>R: 5'- AGTGACACCACCCGTTGT- 3'	218

Table S2 The primer sequence of genes for qPCR

Gene	Sequence of the primer	Length of products (bp)
Caspase-3	F: 5'-TGAGCATGGAAACAATACATGG-3'<br>R: 5'-ATTCTTGGCGAAATTCAAAGG-3'	198
Caspase-8	F: 5'-TCTGCGGACTGGATGTGATT-3'<br>R: 5'-TCTGAGGTTGCTGGTCACAC-3'	165
Caspase-9	F: 5'-TGCCCATACTTCCCGTCC-3'<br>R: 5'-GGTCCAGGTTACCGTCAG-3'	172
Bcl-2	F: 5'-AGGGCATTCAGTGACCTGAC-3'<br>R: 5'-CGATCCGACTCACCAATACC-3'	193
Bax	F: 5'-TGCCTCAGGATGCATCTACC-3'<br>R: 5'-AAGTAGAAAAGCGCGACCAC-3'	199
IL-6	F: 5'-TTCACCTCTCCGGACAAAAC-3'<br>R: 5'-TCTGCCAGTACCTCCTTGCT-3'	122
IL-12	F: 5'-CAGGCCCAGGAATGTTCAAA-3'<br>R: 5'-CGTGGCTAGTTCAAGTGGTAAG-3'	166
IFN-β	F: 5'-GCTAACAAGTGCATCCTCCAAA-3'<br>R: 5'-CCAGGAGCTTCTGACATGCCA-3'	124
IL-1β	F: 5'-AGGGACATGGAGAAGCGATTT-3'<br>R: 5'-TTCTGCTTGAGAGGTGCTGATG-3'	108
METTL3	F: 5'-CCCTATGGGACCCTGACAGA-3'<br>R: 5'-TGACACCAACCAAGCAGTGT-3'	250
METTL14	F: 5'-GGGAGAGTGTGTTTACGCAAG-3'<br>R: 5'-TGAAGTCCCCGTCTGTGCTA-3'	184
WTAP	F: 5'-TCCATTCGTCTTTCCTCTCCG-3'<br>R: 5'-GCCTCACTCAGTCGAACCTTT-3'	130
FTO	F: 5'-GATCTCAATGCCACCCACCA-3'<br>R: 5'-CCACTCAAACTCGACCTCGT-3'	237
ALKBH5	F: 5'-CCCCATCCACATCTTCGAGC-3'<br>R: 5'-CGCATCTAACCTTGTCTTCCTGA-3'	250
YTHDF2	F: 5'-ATGGTAACAAGAGACTGGATGCTG-3'<br>R: 5'-ATGTGTCGCAGTTGGCTATTGG-3'	229
GAPDH	F: 5'-ACATCATCCCTGCTTCTACTGG-3'<br>R: 5'-CTCGGACGCCTGCTTCAC-3'	188

Table S3 Primers for qPCR assay of M gene

Gene	Sequence of the primer	Length of products (bp)
PEDV-M	F: 5'-AGGTCTGCATTCCAGTGCTT-3'<br>R: 5'-GGACATAGAAAGCCCAACCA-3'	216

Table S4 The primer sequence of genes for qPCR

Gene	Sequence of the primer	Length of products (bp)
MMP7	F: 5'-AGGTCTGGCTCATTCGTCTG-3'<br>R: 5'-TCTGGGTTACTTCTCTTTCCGTA-3'	135
CCL2	F: 5'-GTCACCAGCAGCAAGTGTCC-3'<br>R: 5'-CTTGTCCAGGTGGCTTATGG-3'	117
DPP4	F: 5'-GTGACGTGCCTTAGCTGTGA-3'<br>R: 5'-ACCAGGGCCAAAACATCTCA-3'	105
CHAC1	F: 5'-AGCGTTGTGGATTTTCGGGTA-3'<br>R: 5'-ACCACACGACCAGGCATCTT-3'	153
TGFBI	F: 5'-CTGCGGCAAATCAACAGTCA-3'<br>R: 5'-GTTGATCCCACGACTCCCAG-3'	129
SCG2	F: 5'-ATGCAGCAAACCAAAAGCCT-3'<br>R: 5'-CCGGTTTTCAACATCTGGCA-3'	140
SFTA2	F: 5'-GGAATGACTTTGCGACTGA-3'<br>R: 5'-CTGATGGAAGGTGGAGGA-3'	112
ID3	F: 5'-AACCACTGCTACTCGCGTCTCC-3'<br>R: 5'-GAAGGTCGTTTGGTCATTGGAGAT-3'	219
TMEM160	F: 5'-GACAGCCTTCCTTTCCTGGTT-3'<br>R: 5'-CAGCAGGAAGAAGCCATAAGCA-3'	112
GAS6	F: 5'-CCGAGGGTGTCCTGTTCTTC-3'<br>R: 5'-CGCTCAAGCTCCTCAACAGA-3'	178
ACTB	F: 5'-GGATGACGATATTGCTGCGC-3'<br>R: 5'-AGGGTCAGGATGCCTCTCTT-3'	198

Table S5 Primer sequences for MeRIP-qPCR

Gene	Sequence of the primer	Length of products (bp)
GAS6	F: 5'-GACCTCGTCCAGCCCATG-3'<br>R: 5'-TGGTCCCCGCCAACCA-3'	296

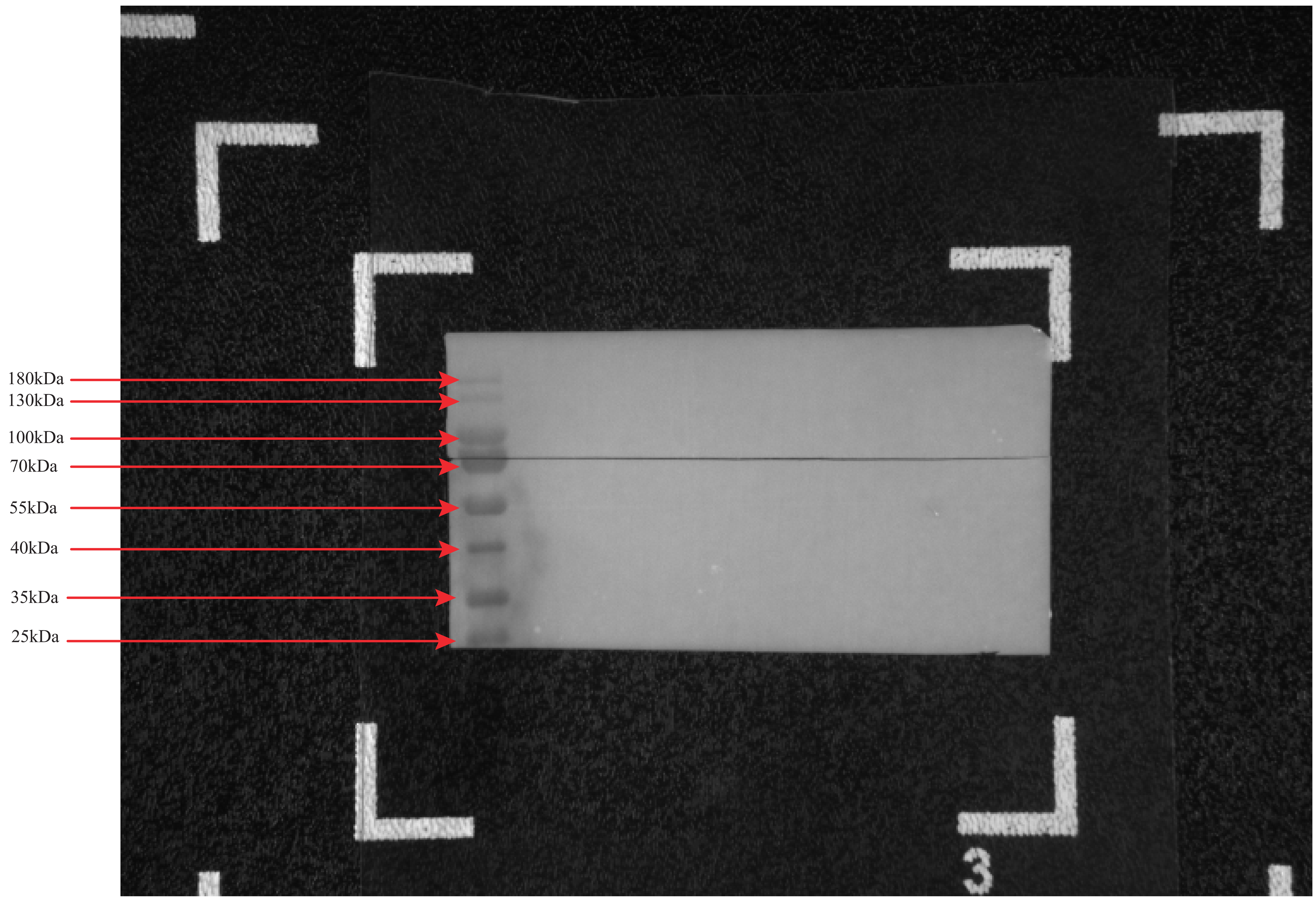

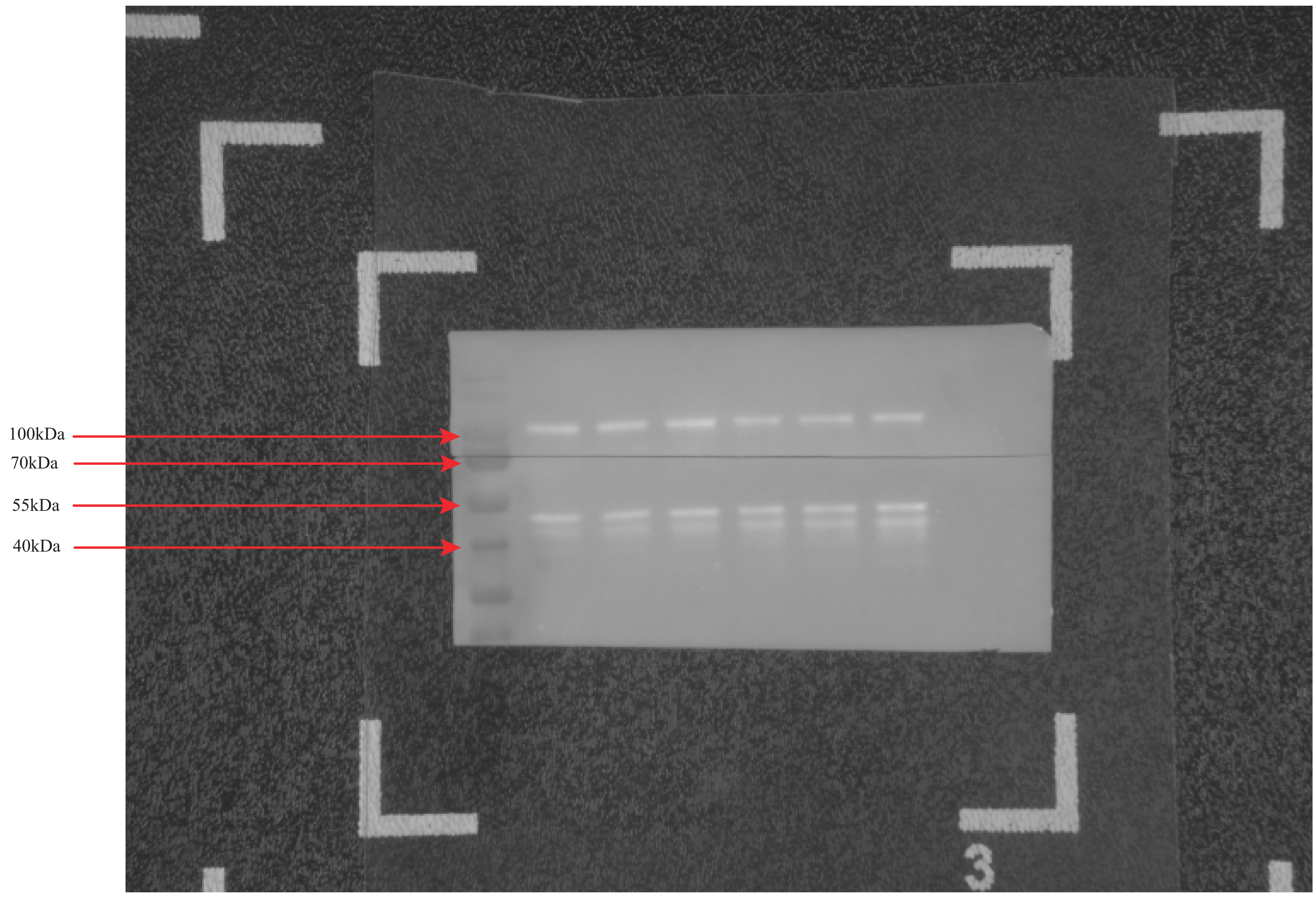

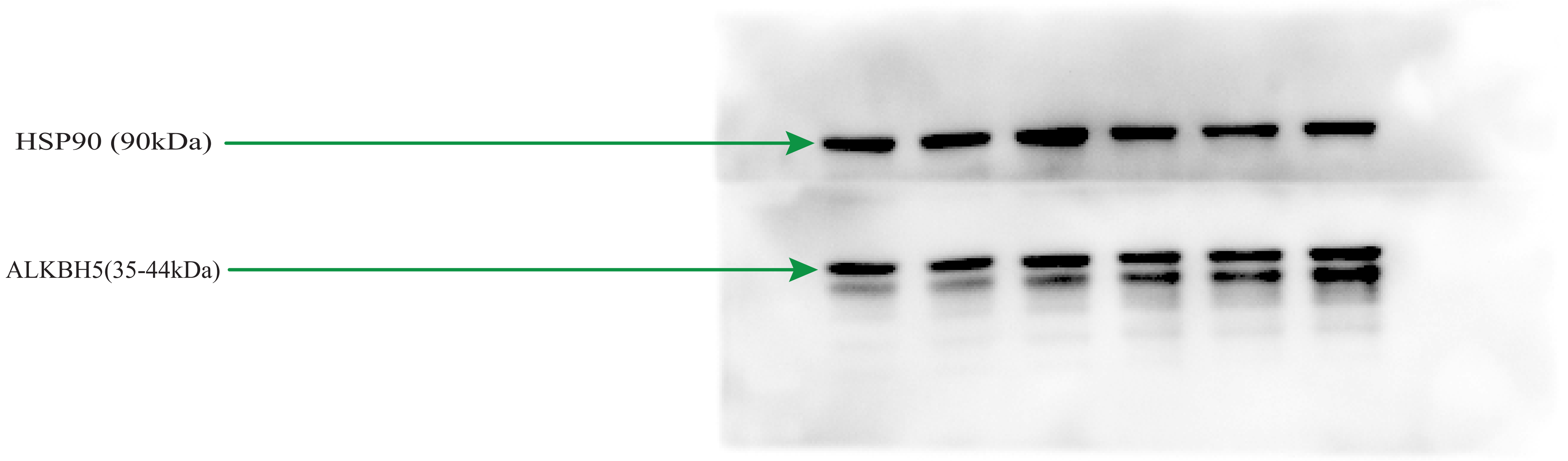

Figure 2f Western blotting for ALKBH5 protein abundance in 3D4/21 cells infected with PEDV for 36 h

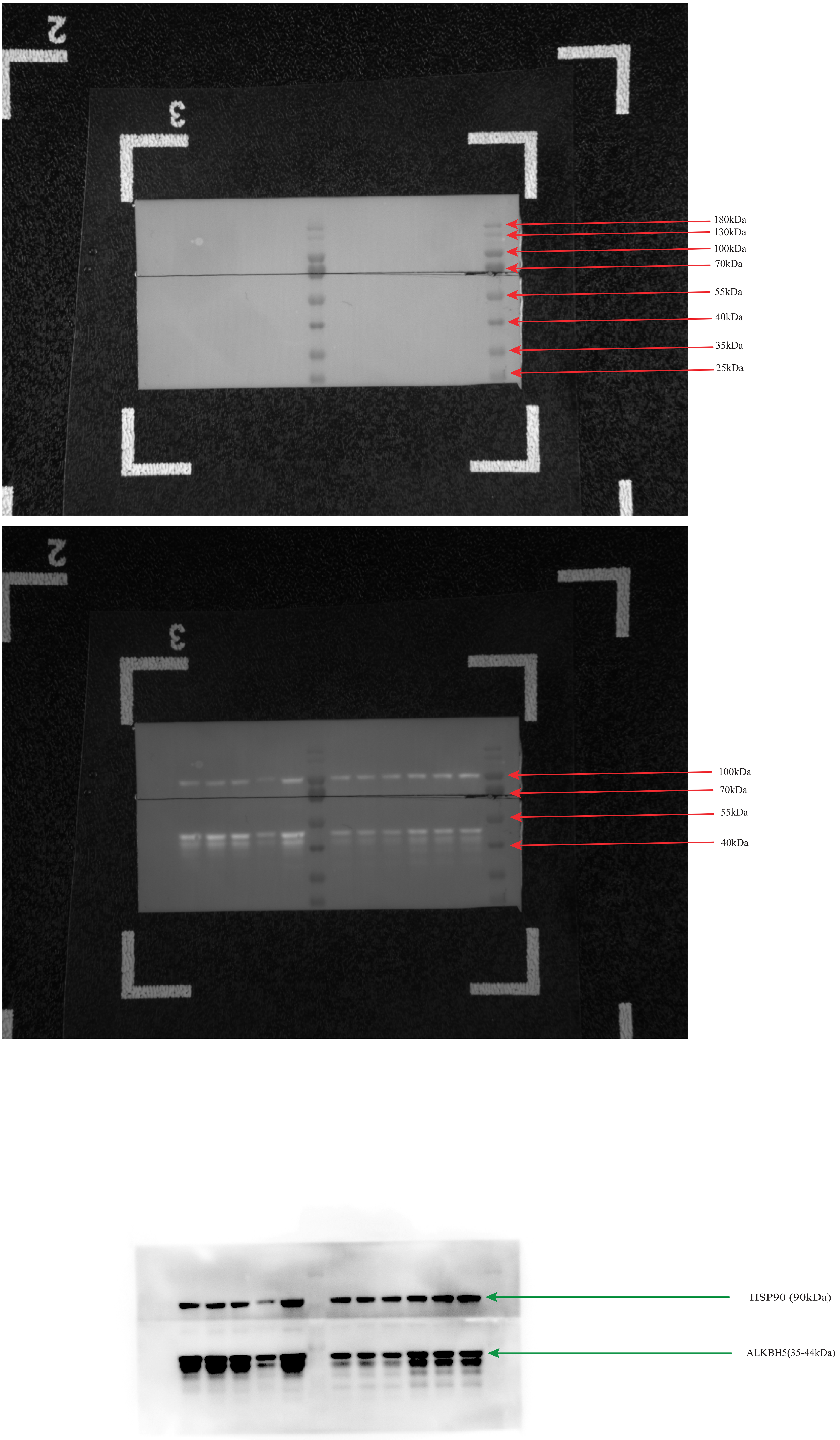

Figure 3c The interference efficiency of ALKBH5 was detected by Western blotting

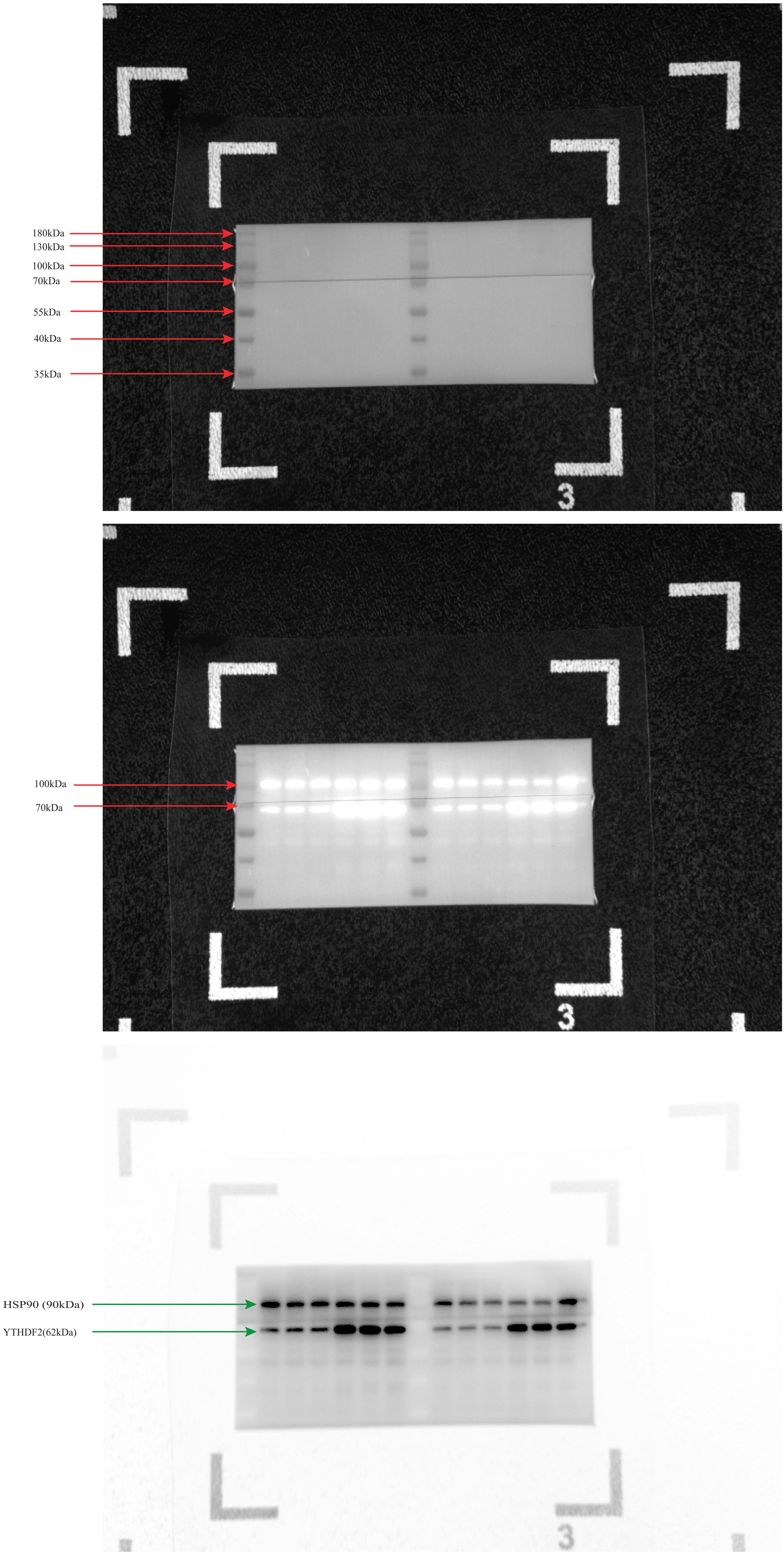

Figure 5i Western blot analysis of *YTHDF2* gene interference efficiency
